# Learning curve for proficiency with a robotic microsurgical system: an *In vitro* study of young medical professionals

**DOI:** 10.1007/s00595-026-03261-9

**Published:** 2026-02-26

**Authors:** Henning Wieker, Tom Michalzik, Dorothee Spille, Juliane Wagner, Jan-Tobias Weitkamp, Jörg Wiltfang, Johannes Spille

**Affiliations:** 1 Department of Oral and Maxillofacial Surgery, Christian Albrechts University, UKSH- Campus Kiel, Kiel, Germany; 2 Department of Neurosurgery, University Hospital Münster, Münster, Germany; 3 Department of Oral and Maxillofacial Surgery, University Hospital of Schleswig-Holstein, Campus Kiel, Arnold-Heller-Straße 3, 24105 Kiel, Germany

**Keywords:** Learning curve, Robotic, Microsurgical, Simulator

## Abstract

**Purpose:**

To evaluate the learning curve and accuracy of microsurgical operation steps achieved by young medical professionals using a microsurgery robotic simulator. The NASA-TLX score was used to assess the workload in the simulation of robotic operation steps for surgical beginners.

**Methods:**

Twenty-three students of dental medicine performed four exercises over 3 consecutive weeks. The exercises consisted of grasping and placing models, grasping and placing a needle, and performing a surgical knot.

**Results:**

There was significant improvement (*p* < 0.05) in the operation time from the first to the second and third measurement points for all exercises. Furthermore, the range of instrument movement became more efficient as the learning curve progressed. The incidence of complications such as instrument collisions improved with each trial. The workload was reduced significantly by the training sessions.

**Conclusion:**

Our study showed that the young medical professionals had a significant learning curve on a microsurgery robotic simulator. The simulator provided a suitable opportunity for students and young professionals to prepare for robotic microsurgery and pique their interest in the field.

## Introduction

In recent decades, robotic surgery has evolved rapidly to become a new standard for many operative procedures, with the rise of the da Vinci system taking minimally invasive methods to a new level of surgery [1]. Furthermore, initial approaches to using robotic systems for reconstructing defects in oral tumor surgery have been made [2]. Sano et al. reported that through the continuous development of these surgical techniques and the advantages of the robotic systems, such as three-dimensional high-resolution images, magnification, and reduction of tremor, robotics could be superior to conventional surgery for some transoral robotic surgery procedures [3]. With the Symani Surgical System^®^ (Medical Microinstruments, MMI, Calci, Italy), a robotic system was developed for microsurgery and supermicrosurgery. Consequently, head and neck surgery now enables plastic reconstruction with vascular and nerve as well as lymph node anastomoses [4].

Young surgeons are particularly interested in new surgical systems, which is why they train in these procedures and strive to use them as frequently as possible [5]. Robotic microsurgery offers a novel surgical approach that can have a major impact on patient outcomes and quality of life, but it requires special skills and capabilities and is often learned through extensive training [6]. Barbon et al. demonstrated that the learning curve for surgeons performing lympho-venous anastomoses with the Symani was very steep, and that the time required to perform the anastomosis has consistently decreased over time [7]. However, the unfamiliar way to use the robotic instruments and the missing tactile feedback require a learning curve that is difficult to overcome, even for experienced surgeons [8]. Therefore, surgical simulation of the Da Vinci system is an excellent opportunity for robotic surgeons to learn and practice their skills in a safe environment [9]. Moreover, it appears that even inexperienced students can handle robotic simulators, experience a steep learning curve, and gain their initial experience in robotic surgery [10]. To date, no simulator training for microsurgery with the Symani has been described in the English literature.

The current study assessed the learning curve and accuracy of microsurgical operation steps by young medical professionals using the Symani. Moreover, the NASA-TLX score was used to assess the workload in the simulation of robotic operation steps for surgical beginners. In this way, new technology can be demonstrated in the education of young professionals, to help establish robotic systems in head and neck surgery, and ultimately increase patient safety in hospitals in the long term.

## Methods

### Study design

**Fig. 1:**
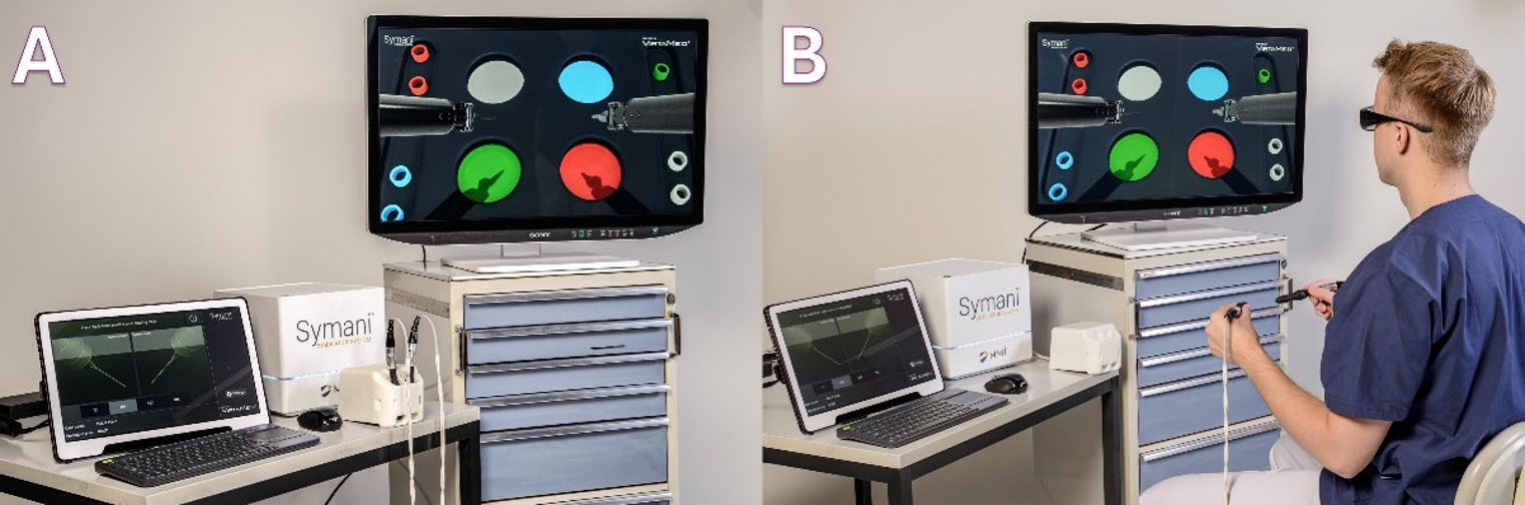
The simulator configuration is shown in **a**). On the left side of the picture is the computer where the individual exercises can be selected and the results displayed. In the centre are the joysticks for using microinstruments in the holder. On the right side is the screen used for tracking the exercises. The image in) shows the training. A student is sitting on a chair and views the exercise on a screen through 3D glasses and controls the 3 mm instruments using the two joysticks and a foot pedal, which is on the floor in front of the chair

The Symani training simulator from MMI was used for this study. The simulator features four exercises that simulate partial steps of microsurgery. As with the original system, the surgeon views a screen with 3D glasses and controls the 3 mm instruments using two joysticks and a foot pedal (Fig. 1). The instruments used were the NanoWrist Needle Holder Suture Cut and the Dilator micro-instruments. In each exercise, the needle holder was held in the right hand, and the dilator was held in the left hand.

Overall, 23 students of dental medicine (7 men and 16 women) participated in the study. None of the students had prior experience in surgical procedures, and all performed their first simulation of a robotic operation. All the young medical professionals received individual instructions before starting the exercises. The study director (J. S.) provided individual instruction to each participant on how to use the simulator. This involved explaining and demonstrating the microsurgery procedures and the use of the fine instruments for each of the four exercises. This instruction lasted approximately 15 min and all questions were answered. Following this, the participants performed the exercises independently, with no further assistance provided, to avoid influencing the individual learning curves.

To describe the learning curve, the students completed the exercises over 3 consecutive weeks. Between these practice periods, the students were able to train independently on the simulator. To avoid influencing the learning curves, the learning time was limited to a maximum of 60 min, and each exercise had a 15-minute time limit. After each training day, the questionnaire “The National Aeronautics and Space Administration Task Load Index” (NASA TLX) was completed to evaluate the individual workload. High values represented a higher workload (values ranged between 0 and 100).

#### Ethical approval

was obtained from the Ethics Commission of the Faculty of Medicine at the Christian-Albrechts-University, Kiel (D 408/24) and was conducted in accordance with the Declaration of Helsinki.

## First exercise

**Fig. 2:**
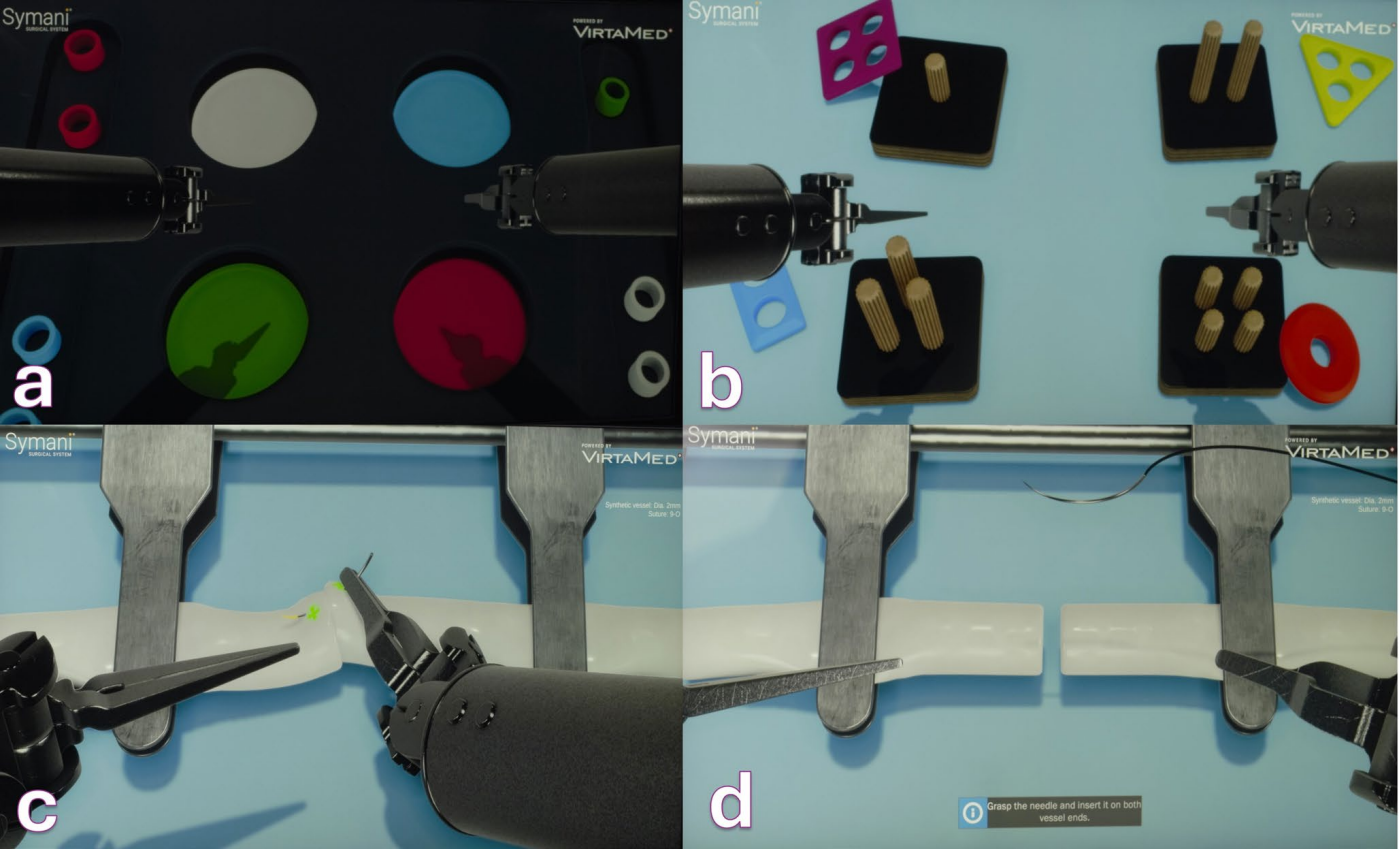
The four exercises are shown. **a**) The coloured objects must be placed in the same-coloured field. **b**) The pieces must be placed in the corresponding field. **c**) The needle must be inserted through the yellow fields of the anastomosis. **d**) The needle must be inserted through both ends of the anastomosis, and a surgical knot must be tied

There were four colour-marked fields (green, red, blue, white) of equal size, within which two coloured cones had to be placed, so that they were in the correct colored field (Fig. 2a). Both the needle holder and the dilator could be used to hold the figures, which were about 2 mm in size. The figures were not rigid, but somewhat flexible to simulate moving human tissue. The colour-marked fields were approximately 5 mm in size.

## Second exercise

There were four different shapes with one, two, three, and four holes, approximately 1 mm in size. These shapes had to be placed on a device that fits the holes (Fig. 2b). The materials were rigid, but the shapes and devices were movable. This simulated the handling of rigid structures, such as the medical devices used, like stents, or bony structures in the human anatomy.

## Third exercise

A 9.0 sized needle had to be grasped then pierced through two vessels at predetermined points. Thus, as with an anastomosis, one vessel had to be pierced from the outside inwards and the other vessel from the inside outwards. The vessel had a diameter of approximately 2 mm (Fig. 2c).

## Fourth exercise

A single button suture, as with an anastomosis, had to be performed. A 9.0 sized needle had to be grasped, then pierced through two vessels with a diameter of approximately 2 mm. The exercise was finished when a knot was tied and cut off (Fig. 2d).

### Evaluation of the training simulation

The simulator collected a lot of data on its own. For evaluating the learning curve, we measured:

- Operation time (all exercises).
- Extension of instrument movements (all exercises).
- Number of needle stitches (exercise three).
- Collision of the instruments (exercise four).

### Statistical analysis

Statistical analyses were performed using SPSS (IBM^®^, Ehningen, Germany). Normally distributed and non-normally distributed continuous variables are expressed as mean (± SD), and categorical variables are presented as total counts. The progress of the individual exercises is described as a learning curve at different time points and was calculated by an analysis of variance (ANOVA). Associations were considered significant when the p-value was < 0.05.

## Results

**Fig. 3:**
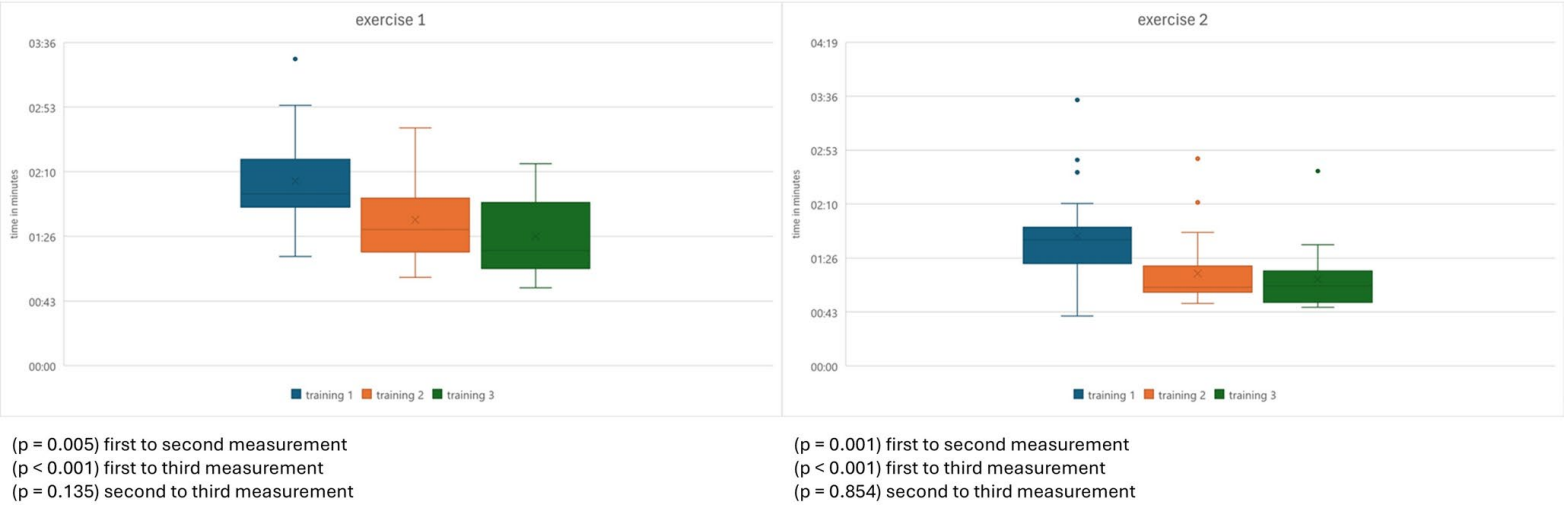
The measured values and p-values for the first and second exercises are shown in box plots

**Fig. 4:**
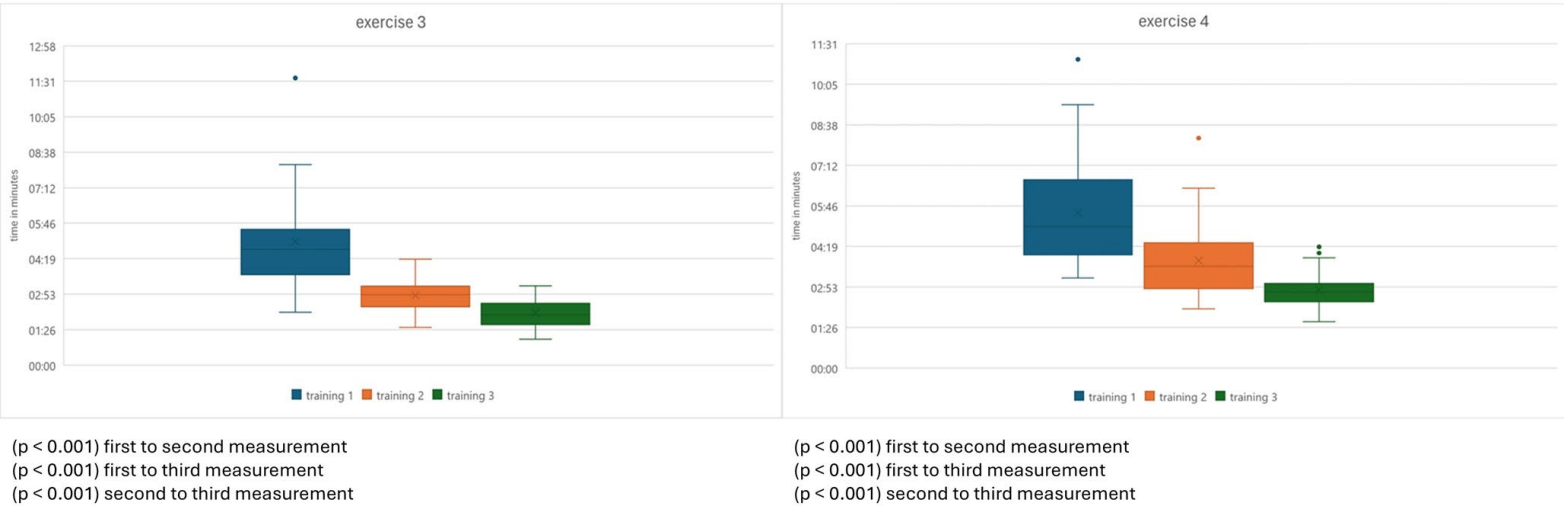
The measured values and p-values for the third and fourth exercises are shown in box plots

Table 1 shows the mean times and standard deviation for each exercise. Figures 3 and 4 show the box plots. One student was unable to complete the third exercise at the first measurement point within the 15-minute time limit, but at the second and third measurement points, the time limits were significantly undercut for all students. Each study participant trained between periods, but this varied considerably, ranging from 15 to 60 min. Some students trained immediately after each period, while others practiced on a different day.

**Table 1:** Mean values and standard deviations (SD) of the operation times for the four exercises

	Exercise 1	Exercise 2	Exercise 3	Exercise 4
Measurement time point 1	02:03 ± 00:35 min	01:43 ± 00:36 min	05:27 ± 02:54 min	05:31 ± 02:03 min
Measurement time point 2	01:37 ± 00:28 min	01:14 ± 00:27 min	02:58 ± 00:57 min	03:49 ± 01:27 min
Measurement time point 3	01:26 ± 00:26 min	01:09 ± 00:24 min	02:15 ± 00:51 min	02:46 ± 00:45 min

### First exercise

The average exercise time was 2:03 min at the first measurement and 1:26 min at the third measurement. There was a significant improvement from the first to the second measurement time point (*p* = 0.005) as well as from the first to the third measurement time point (*p* < 0.001). The improvement from the second and third measurement time points was not significant (*p* = 0.135). The average extensions of instrument movements were 43.2 millimetres for the left hand and 52.7 millimetres for the right hand at the first measurement time, and 38 millimetres for the left hand and 40.2 millimetres for the right hand at the third measurement time.

### Second exercise

The average exercise time was 1:43 min at the first measurement and 1:09 min at the third measurement. There was a significant improvement from the first to the second measurement time point (*p* = 0.001) as well as from the first to the third measurement time point (*p* < 0.001). The improvement from the second and third measurement time points was not significant (*p* = 0.854). The average extensions of instrument movements were 20.6 millimetres for the left hand and 30.2 millimetres for the right hand at the first measurement time, and 19 millimetres for the left hand and 23.3 millimetres for the right hand at the third measurement time.

### Third exercise

The average exercise time was 5:27 min at the first measurement and 2:15 min at the third measurement. There was a significant improvement from the first to the second measurement time point (*p* < 0.001) as well as from the first to the third measurement time point (*p* < 0.001), and from the second to the third measurement time point (*p* < 0.001). The average extensions of instrument movements were 22.8 millimetres for the left hand and 16.1 millimetres for the right hand at the first measurement time, and 10.9 millimetres for the left hand and 6.7 millimetres for the right hand at the third measurement time. The number of needle punctures was 11.36 at the first measurement time point, 10.09 at the second, and 8.41 at the third.

### Fourth exercise

The average exercise time was 5:31 min at the first measurement and 2:46 min at the third measurement. There was a significant improvement from the first to the second measurement time point (*p* < 0.001) as well as from the first to the third measurement time point (*p* < 0.001), and from the second to the third measurement time point (*p* < 0.001). The average extensions of instrument movements were 33.5 millimetres for the left hand and 37.5 millimetres for the right hand at the first measurement time, and 22.4 millimetres for the left hand and 19.5 millimetres for the right hand at the third measurement time. The number of collisions of the instruments was 12.52 at the first measurement time point, 8.48 at the second, and 4.78 at the third. There was a significant improvement from the first to the third measurement time point (*p* = 0.001), but no significant difference between the first and second measurement time points (*p* = 0.068). There was also a significant improvement from the second to the third measurement time point (*p* = 0.008).

### NASA TLX

The average workload was 48.17 (± 9.59) at the first measurement time point, 40.4 (± 11.95) at the second measurement time point, and 33.64 (± 14.75) at the third measurement time point. Significance was calculated for the first to the second measurement time point (*p* = 0.02) as well as for the first to the third measurement time point (*p* < 0.001).

## Discussion

This study demonstrated that young medical professionals without prior surgical experience can operate a robotic simulator for microsurgery immediately and can overcome a steep learning curve. A significant improvement in time was observed for all four exercises. This included grasping and placing objects, handling a needle, and knotting when simulating an anastomosis suture. Moreover, the handling and collision rates improved quickly and significantly. Kang et al. reported similar results for medical students performing vesicourethral anastomosis. The mean time, collision, and critical errors were significantly reduced, although more repetitions were performed, and a safe anastomosis was first achieved after an average of 74 attempts [10]. Lerner et al. described that after four training sessions, the use of a simulator in residents’ and students’ training can improve surgical skills significantly [11]. These results support our observation that only a few repetitions on the simulator are necessary to improve surgical skills significantly. However, this does not mean that surgery could be performed safely by inexperienced surgeons on patients with more difficult conditions, such as bleeding or movement of the situs. Inexperienced surgeons must practice frequently on the simulator before attempting to operate.

Yu et al. reported finding that the simulation of robotic thyroidectomy improved the skills of young medical professionals from trial to trial [12]. In our study, the students’ skills in performing easy exercises attempt such as grasping and placement improved slightly from the second to the third, but their skills in performing more complex exercises such as handling the needle and knotting the anastomosis improved significantly. Simulation programs can be repeated continually to ensure that even inexperienced surgeons gain confidence in serious operations, thereby reducing morbidity and adverse event rates [13]. In this context, it should be mentioned that students are less experienced in simulation exercises than young doctors, but frequent training can close this gap [14].

Robotic surgical procedures are being performed increasingly worldwide, and training modules on simulators or models can improve the surgical skills of both inexperienced and experienced surgeons, and provide training in rare and complex operations [15–17]. Inexperienced and experienced surgeons can expand their surgical repertoire, and life-threatening complications can be avoided [18]. Moreover, simulator exercises can provide objective information on using the robotic surgical system proficiently and indicate surgical experience, as well as whether an operation can be performed safely on a patient [19]. Stefanidis et al. found that students achieve good results by using the robot and even prefer it to laparoscopy [20]. This highlights the importance of new techniques, particularly for the younger generation of surgeons and suggests that these techniques will continue to have a significant impact on surgical procedures in the future.

In our study, the time between exercises was set at 1 week, to allow students to assimilate what they had learned. Kang et al. showed that training on consecutive days achieves the most effective results; however, intensive training on just 1 day does not seem sensible [21]. Inexperienced surgeons appear to retain their surgical skills after regular training sessions and then continue to perform well on the simulator. This increases confidence and mastery of robotic surgical tasks [22]. After this training, young doctors seem to perform their first operations more safely and with fewer complications [23]. Furthermore, the students showed a significant reduction in workload during the exercises. While the NASA TLX evaluation still indicates some workload during the exercises, suggesting that high stress levels may exist for operations on patients, the importance of simulator training is evident, as these stresses can be mitigated and significantly reduced through training. Without training, a high stress level can be expected. Bell et al. even recommend using the NASA TLX as a measuring instrument for research and teaching settings to score surgical difficulty and monitor a trainee’s proficiency over time [24]. The NASA TLX can also be helpful for experienced robotic surgeons to measure and evaluate their workload objectively and effectively [25].

Robotic microsurgery is a relatively new surgical field and requires much experience before surgical steps can be carried out quickly and safely. However, Rabbin-Birnbaum et al. were able to show that the learning curve for robotic microanastomoses is short and encouraging [26]. Robotic surgery appears to require more time than conventional suturing [25], which is why preoperative training to learn the skills and capitalize on the learning curve is beneficial. It is essential to identify which surgical procedures are suitable for the robot and to what extent this new technology can enhance everyday operations, making them easier and safer [27]. Furthermore, training on laboratory models, animals, and cadavers should be considered, although this level of training is associated with ethical concerns and high costs [28]. Training on simulators is easier to implement and doctors can practice at any time.

The current study has some limitations. First, the lab conditions cannot be compared with real life: the conditions are relatively simple, and no blood or movement of the site can be observed during the simulation. Second, this study involved a small sample of students and a limited number of exercises. A larger number of subjects is necessary to confirm the steep learning curve of inexperienced surgeons. The variability in practice time among students is another important factor that may have affected the study results. An exact specification of the practice time would be useful. In the future, a large database or a prospective in vivo study for microsurgery, involving both experienced and inexperienced surgeons, should be conducted.

## Conclusions

The findings of this study showed that training on a robotic simulator could be an effective alternative for learning surgical robotic skills. Inexperienced surgeons and medical students can practice straightforward and complex surgical steps several times until they feel confident about performing their first operations on patients. Furthermore, it enables the objective assessment of a surgeon’s skill in maneuvering a technique and whether an operation is feasible given the individual’s level of training. Overall, students show a steep learning curve in microsurgery simulation and quickly become familiar with the novel surgical system. The simulator could provide a suitable opportunity for students and young medical professionals to prepare for robotic microsurgery and pique their interest in this field.

## Data Availability

The data that support the findings of this study are available from the corresponding author upon reasonable request.
